# Perceived Usability, User Experience, and Technology Acceptance of Role-Specific Augmented Reality Decision Support Tools for Cardiac Arrest Resuscitation: Prospective Observational Pilot Study

**DOI:** 10.2196/72013

**Published:** 2026-04-07

**Authors:** Ryan Kang, Adam Cheng, Yiqun Lin, Hyeongil Nam, Jennifer Davidson, Donovan Curtis Duncan, Johan N Siebert, Sergio Manzano, Alexandre De Masi, Ana Rajic, Sharleen Kayne Olanka, Frederic Ehrler, Kangsoo Kim

**Affiliations:** 1 Department of Electrical and Software Engineering, Schulich School of Engineering, University of Calgary, 2500 University Drive NW, Calgary, AB, T2N 1N4, Canada, 1 403-220-5644; 2 KidSIM Simulation Program, Alberta Children's Hospital, Calgary, AB, Canada; 3 Department of Pediatrics and Emergency Medicine, Cumming School of Medicine, University of Calgary, Calgary, AB, Canada; 4 Department of Pediatric Emergency Medicine, Geneva University Hospitals, Geneva, Switzerland; 5 Faculty of Medicine, University of Geneva, Geneva, Switzerland; 6 Educational Technologies and Learning Sciences (TECFA), Faculty of Psychology and Educational Sciences, University of Geneva, Geneva, Switzerland; 7 Division of Computer Sciences, Geneva University Hospitals, Geneva, Switzerland

**Keywords:** cardiopulmonary resuscitation, augmented reality, simulation training, wearable electronic devices, digital health, user-computer interface, decision support systems, clinical guideline adherence, technology acceptance, user-centered design

## Abstract

**Background:**

Cardiac arrest is a critical medical emergency that requires strict adherence to clinical guidelines to achieve optimal outcomes. Deviations from these guidelines, often due to task complexity, can adversely affect patient outcomes. Augmented reality (AR) offers a way to deliver role-specific, in-view guidance, but evidence on its perceived usability, user experience, and acceptability in cardiac arrest resuscitation remains limited.

**Objective:**

This study aimed to design, develop, and evaluate a role-specific AR decision support system for resuscitation team leaders and medication nurses. In this observational study, we assessed clinicians’ perceived usability, user experience, and technology acceptance of the new AR system in a high-fidelity simulated cardiac arrest scenario.

**Methods:**

We conducted a prospective observational pilot study using a high-fidelity simulated pediatric cardiac arrest scenario. A total of 10 clinicians were recruited from Alberta Children’s Hospital, including 5 (50%) of 10 pediatric emergency physicians serving as team leaders (men: 3/5, 60%, and women: 2/5, 40%; median age 41, IQR: 40-42 y) and 5 (50%) of 10 emergency nurses serving as medication nurses (men: 1/5, 20%, and women: 4/5, 80%; median age 45, IQR: 42-46 y). Participants used role-specific AR decision support interfaces deployed on HoloLens 2 head-mounted displays. Following the simulation, perceived usability, user experience, and technology acceptance were assessed using validated questionnaires: the System Usability Scale, User Experience Questionnaire, and Technology Acceptance Model. Data were collected via postsimulation surveys and analyzed descriptively.

**Results:**

Descriptive analyses were performed without inferential statistical testing. The mean System Usability Scale scores were 75.5 (SD 9.25, 95% CI 64.0‐87.0) for team leaders and 82.0 (SD 11.20, 95% CI 68.0‐96.0) for medication nurses. User experience was positive across roles, with mean User Experience Questionnaire scores indicating favorable attractiveness (team leaders: 1.87, SD 1.14, 95% CI 0.45‐3.28; medication nurses: 2.43, SD 0.52, 95% CI 1.79‐3.08), pragmatic quality (team leaders: 1.88, SD 0.87, 95% CI 0.80‐2.97; medication nurses: 1.80, SD 0.69, 95% CI 0.94‐2.66), and hedonic quality (team leaders: 2.40, SD 0.89, 95% CI 1.30‐3.50; medication nurses: 2.28, SD 0.69, 95% CI 1.42‐3.13). Technology acceptance was high, with mean combined Technology Acceptance Model scores of 5.92 (SD 0.46, 95% CI 5.35‐6.49) for team leaders and 6.02 (SD 0.56, 95% CI 5.32‐6.71) for medication nurses.

**Conclusions:**

This study introduces a novel role-specific AR decision support system that delivers tailored, in-view guidance to resuscitation team leaders and medication nurses during cardiac arrest. Unlike prior cognitive aids that present uniform or device-agnostic information, this system explicitly adapts interface content and structure to distinct clinical roles and workflows. The findings contribute early empirical evidence on the perceived usability, user experience, and acceptability of role-tailored AR support in high-acuity team settings and yield transferable design principles for developing role-aware AR interfaces. In real-world contexts, such systems may support protocol adherence and team coordination during resuscitation training and early-stage clinical deployment, informing future evaluations that incorporate objective performance and workflow outcomes.

## Introduction

Cardiopulmonary resuscitation (CPR) is administered to thousands of patients experiencing cardiac arrests (CAs) each year in North America [1]. Guideline-compliant basic life support and advanced life support guidelines significantly improve patient outcomes following CA [2,3]. However, health care providers often face challenges in consistently adhering to these guidelines during in-hospital CA events. Deviations, such as delays in epinephrine administration, defibrillation, and medication dosing errors, are commonly linked to poor patient outcomes [4]. These deviations are often attributed to the high cognitive demands and mental workload experienced by resuscitation team members [5,6].

Cognitive aids, designed to assist in decision-making and information recall, have demonstrated improved adherence to resuscitation guidelines during simulated cardiopulmonary arrest events [7-11]. By reducing errors and improving the timing of key interventions, cognitive aids can enhance clinical performance [12]. However, traditional cognitive aids, such as pocket cards, sometimes introduce delays in initiating CPR or administering drugs due to their design limitations or complexity, highlighting the need for more efficient, role-specific decision-support solutions. Recent scoping and systematic reviews published in the past few years highlight a growing interest in immersive technologies, including augmented reality (AR), for resuscitation training and emergency care, while also identifying variability in system design, evaluation approaches, and integration with clinical workflows [13,14].

AR overlays digital content onto the physical environment, enabling real-time delivery of context- and role-specific prompts directly in the user’s field of view [15]. AR systems have been explored in CPR and emergency care training contexts, with some evidence of improved engagement and task performance compared with conventional approaches, although results remain heterogeneous and context-dependent [16,17]. Previous AR-based work in resuscitation and safety-critical domains further suggests that spatially registered visual cues can support situational awareness and reduce reliance on external reference materials during time-sensitive tasks [15,18,19]. Despite this growing body of work, recent reviews emphasize that evidence regarding the usability, user experience, and acceptability of wearable AR systems in CA resuscitation—particularly from the perspective of end users—remains limited [13,14].

To address these gaps, this study presents the design, development, and formative evaluation of an AR-based decision support system tailored to the resuscitation team leader (physician) and medication nurse roles during CA resuscitation. The objectives of this study were to (1) describe how team leaders and medication nurses perceive the AR system’s usability and user experience when used during a simulated resuscitation scenario and (2) describe how team leaders and medication nurses perceive the system’s acceptability and its potential for future integration into clinical practice.

## Methods

### Ethical Considerations

Ethics approval was obtained from the University of Calgary (REB23-1007) and the University of Geneva Health Research Ethics Boards (Req-2023‐00162). Before participation, all participants were provided with written information describing the study purpose, procedures, potential risks, and data handling practices, and written informed consent was obtained. Participation was voluntary, and participants were informed that they could withdraw from the study at any time without consequence. Consent included permission to collect survey data and to use nonidentifiable data and images generated during the simulation for research and publication purposes. Privacy and confidentiality were ensured for all study participants. No images included in the manuscript or supplementary materials contain identifiable information about individual participants. Participants did not receive any compensation for their role in this study.

### Study Design: Experimental Setting

This study was designed as a prospective observational pilot study conducted in a high-fidelity pediatric CA simulation setting. The following section describes the overall process of the AR system design and development, which was used in the study.

#### Iterative Design and Development Process of the AR System

**Figure 1.**
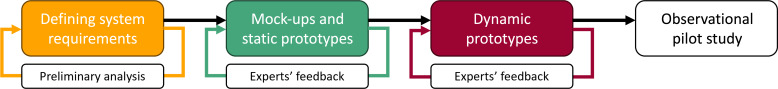
Four-phase iterative development process.

For our study, role-specific AR decision support systems for team leaders (physicians) and medication nurses were developed following a 4-phase, iterative prototyping process grounded in user-centered and clinician-informed design practices (Figure 1). Phases 1 to 3 focused on system design and development, whereas phase 4 evaluated the final prototype in a simulation-based clinical environment through an observational pilot study. The objective of this process was to progressively refine AR design concepts into a stable, simulation-ready system through iterative feedback and close collaboration with clinical domain experts.

#### Phase 1: Defining System Requirements

This phase focused on identifying the clinical, informational, and workflow requirements necessary to guide the content and design of the role-specific AR interfaces. A total of 30 health care professionals (15, 50%, emergency physicians and 15, 50%, emergency nurses) from Alberta Children’s Hospital (ACH) and Geneva University Hospitals were surveyed to assess preferences for role-specific information, AR layout components, timer placement and behavior, and medication-related display features. Clinicians viewed role-specific, task-focused information as important elements of the AR system. Both physicians and nurses emphasized the utility of receiving targeted, step-relevant prompts through the AR headset. Real-time updates regarding current and upcoming tasks (“next steps”) were perceived to enhance workflow by reducing the need to reference external materials visually. Both groups rated time-based cues highly, with the integration of a CPR timer (for the team leader) and an epinephrine timer (for the team leader and medication nurse) described as highly important for the AR headset. A detailed list of resuscitation medications and associated dosages was also rated highly for both groups of providers. Physicians expressed the desire to be notified when medications were given. These insights directly informed the layout, information hierarchy, and alerting behavior of the static (phase 2) and dynamic (phase 3) prototypes, ensuring that the AR interface design aligned with clinicians’ informational needs and workflow demands. Table 1 provides a summary of key insights from phase 1.

**Table 1.** Key insights from each phase of the iterative development process.

Phase	Key insights
Phase 1: Defining system requirements	Role-specific, task-focused clinical prompts Current tasks (prioritized) Next steps (prioritized) CPR^a^ timer (team leader) Epinephrine timer (team leader and medication nurse) Medication reference Medication given—notification for team leader
Phase 2: Mock-ups and static prototypes	Interface separation by user role improves clarity and relevance of displayed information. Visually simple, structured layouts are preferred for rapid information recognition. Timers and other time-sensitive elements should be placed in the upper peripheral field of view to avoid obstructing the patient. Current tasks should be listed on the left and next steps to the right. Cardiac rhythm should be displayed in the physician’s augmented reality headset. Patient weight should be displayed on the medication nurse display. Other UI^b^ elements should be fixed in space (e.g., clinical algorithm, Hs and Ts, medication reference) to avoid interference when team members move through the field of view.
Phase 3: Dynamic prototype	Functional CPR and epinephrine timers included escalating visual cues. The UI was visually refined with higher contrast, low-profile components, reorganized medication content, and larger fonts. Medication card (team leader): categorized drug details with interactive dose counters Guideline algorithm panel (team leader): full cardiac arrest algorithm visualization with a stage-tracking arrow. Hs and Ts reference (team leader): a structured list of reversible causes for rapid diagnostic review. Medication card (medication nurse): categorized drugs with strength, dose, volume, and instructions, plus an interactive syringe counter for tracking prepared or administered doses. UI elements were arranged to maximize visibility and minimize occlusion during dynamic resuscitation.

a CPR: cardiopulmonary resuscitation.

b UI: user interface.

#### Phase 2: Mock-Ups and Static Prototypes

In this phase, the requirements identified in phase 1 were transformed into static prototype designs. Initial mock-ups were created to visualize the AR layout, role-specific information elements, and overall display functionality. To optimize role-specific design, separate static layouts were developed for the team leader and medication nurse roles. The team leader interface focused on 4 key elements (i.e., the CPR timer, epinephrine timer, current task list, and next task list), whereas the medication nurse interface incorporated 3 core elements (i.e., epinephrine timer, current task list, and next task list). A total of 9 static prototypes were created for the team leader, and 5 prototypes were created for the medication nurse, exploring variations in spatial arrangement and visual hierarchy (Figures S1 and S2 in Multimedia Appendix 1).

In total, 5 emergency room physicians and 5 emergency room nurses from ACH were selected to provide feedback on static layouts for their corresponding profession. Participants were shown each static layout in sequence and asked to provide verbal feedback regarding spatial organization, information grouping, font and icon size, color and contrast of user interface (UI) elements, and the position of UI elements relative to equipment and providers in the clinical space. Physicians rated their top 3 display options, and nurses were asked to rate their top 2 options. Feedback was documented using annotated screenshots and meeting notes. On the basis of this feedback, 3 dynamic prototypes for the team leader and 2 dynamic prototypes for the medication nurse were developed to ensure that UI elements were easy to identify, accessible, and minimally intrusive within the AR field of view. Table 1 provides a summary of key insights from phase 2.

#### Phase 3: Dynamic Prototypes

This phase involved developing and iteratively refining dynamic AR prototypes for both the team leader and medication nurse roles. Dynamic interface layouts were implemented using Unity and deployed on the Microsoft HoloLens 2. The initial dynamic versions preserved the core components established during the static prototyping phase, while introducing functional timers, refined visual elements, and interactive components. For the medication nurse interface, an adjustable epinephrine dose counter was implemented, allowing users to adjust the number of doses prepared or administered. To guide iterative refinement, the 10 participants from phase 2 returned to provide feedback on the dynamic prototypes. Participants were asked to evaluate layout preferences, timer behavior, visual clarity, and ease of interaction. Additional role-specific questions were directed to team leaders and medication nurses to capture feedback aligned with each role’s clinical responsibilities.

**Figure 2.**
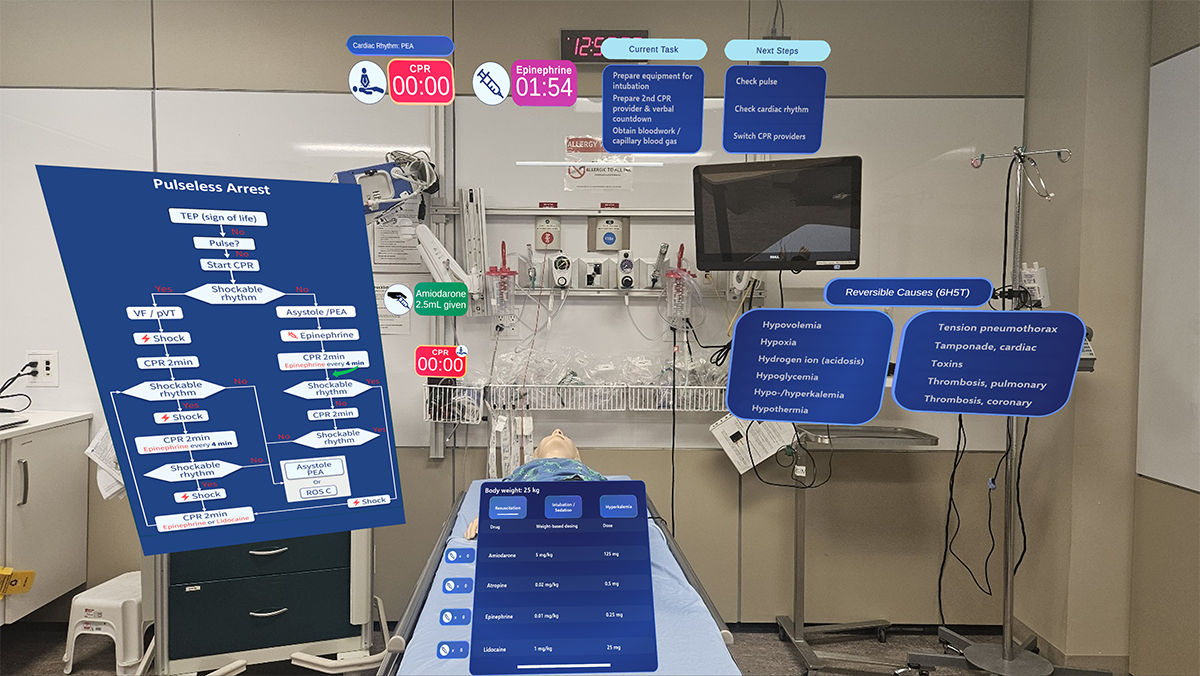
Team leader display showcasing real-time CPR and medication timers, visual alerts for task progression, and stepwise guidance for current and upcoming guideline tasks during a simulated cardiac arrest (CA) scenario. When in use, the CA algorithm, reversible causes, and medication card are positioned out of view when the team leader is looking straight ahead. To view each of these items, the team leader must turn to the left (to see the algorithm), to the right (to see the reversible causes), or look slightly down (to see the medication card). CPR: cardiopulmonary resuscitation; PEA: pulseless electrical activity; pVT: pulseless ventricular tachycardia; ROSC: return of spontaneous circulation; TEP: Treatment Escalation Plan; VF: ventricular fibrillation.

**Figure 3.**
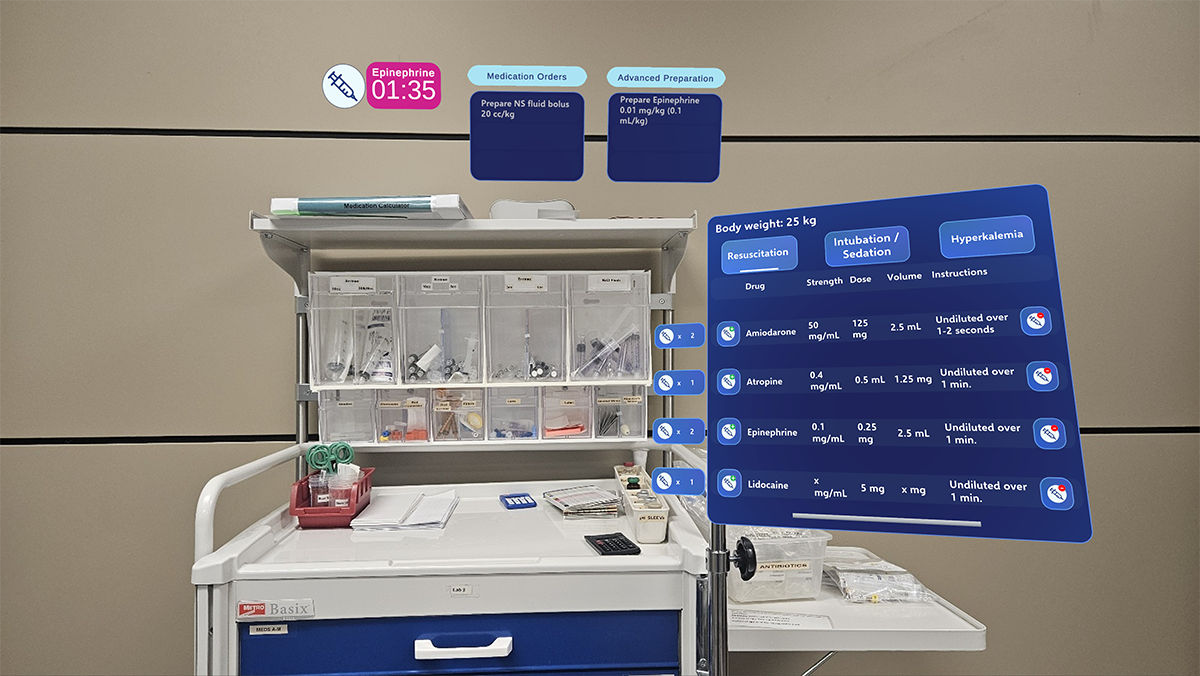
Medication nurse display showing step-by-step guidance on drug dosages, preparations, and administration timing, with a real-time epinephrine timer for ensuring timely interventions.

Feedback sessions identified refinements to visual hierarchy, timer behavior, text sizing, and the placement of role-specific components. Experts also evaluated the positioning of fixed elements such as the CA algorithms, reversible causes (Hs and Ts), and the medication card, providing insight into potential visual obstruction during active resuscitation. Feedback informed key improvements to support clarity, usability, and workflow alignment. Functional CPR and epinephrine timers were revised to include escalating visual cues (yellow flash at 10 s and rapid red flash at 1 s). The UI was visually refined with higher contrast, low-profile components, reorganized medication content, and larger fonts. An interactive epinephrine dose counter was added for medication nurses. Participants also emphasized the need to reposition or hide large reference panels to prevent obstruction and maintain clear grouping of current and upcoming tasks. Table 1 provides a summary of key insights from phase 3. All recommended changes were incorporated into one final updated dynamic prototype for the team leader (Figure 2) and medication nurse (Figure 3).

#### Final System Architecture and Components

The AR system used in this study was developed using a server-client architecture to enable seamless, real-time synchronization between a web-based control system operated by the experimenter and the role-specific AR interfaces used by the team leader and medication nurse (Figure S3 in Multimedia Appendix 1). This architecture ensured that each user received only the information relevant to their role while maintaining consistent timing, event updates, and algorithm progression across devices.

- Web-based control system (server): A centralized web-based control system was implemented to manage scenario flow and synchronize data to both AR devices (Figure S4 in Multimedia Appendix 1). During the simulation, the experimenter used this interface to advance the CA algorithm, trigger event notifications, reset timers, and record medication administration (Figure S5 in Multimedia Appendix 1). All adjustments made on the server were immediately transmitted to the AR clients, enabling real-time display without perceptible delay.
- AR interfaces for team leader and medication nurse (client): Two separate AR client applications were deployed on the HoloLens 2 devices, one for each role. These interfaces displayed synchronized timers, role-specific prompts, algorithm guidance, medication information, and interactive elements (e.g., dose counters). The client applications integrated incremental refinements derived from clinician feedback during dynamic prototyping, ensuring that the displays aligned with each role’s workflow and cognitive demands.

Together, the server-client architecture, real-time synchronization, and role-specific display features formed a cohesive system for supporting resuscitation team members during high-acuity pediatric CA scenarios.

#### Phase 4: Simulation-Based System Evaluation

Phase 4 consisted of a prospective, observational pilot study in which participants managed a simulated CA scenario using the final prototype of the AR system.

### Participants and Sample Size

Participants were recruited from the pediatric emergency department at ACH. All participants had completed basic life support and pediatric advanced life support training. There were no specific exclusion criteria. A convenience sample of 10 health care professionals participated, consisting of 5 (50%) pediatric emergency physicians (team leaders) and 5 (50%) emergency nurses (medication nurses). The same 10 participants who provided feedback in phases 2 and 3 were paired into physician-nurse dyads, with each clinician assigned the AR interface corresponding to their respective profession.

### Study Procedure: Simulated CA Scenario

The simulation scenarios took place in the KidSIM Pediatric Simulation Center at ACH using a high-fidelity pediatric manikin (Laerdal SimJunior). Each dyad (1 physician team leader and 1 medication nurse) was embedded within a larger clinical resuscitation team composed of 3 additional research actors playing the roles of airway provider, bedside clinician, and CPR provider to recreate an authentic team-based resuscitation environment. The 2 study participants wore HoloLens 2 devices displaying their respective role-specific AR interfaces.

The scenario simulated an in-hospital pediatric CA involving a 5-year-old boy who presented with pulseless ventricular tachycardia, progressing through ventricular fibrillation and pulseless electrical activity, before achieving return of spontaneous circulation at the 18-minute mark. Participants, acting as team leader or medication nurse, were guided by visual prompts on their respective AR displays. The team leader guided overall clinical management, including airway management, CPR, defibrillation, and ordering medications. The medication nurse handled medication preparation and administration, following role-specific cues on the AR interface. Research actors were trained to function in their role as they would in a real CA.

### Measures

To provide a comprehensive assessment of the AR support system’s perceived usability, user experience, and acceptance, we used 3 well-established instruments. The System Usability Scale (SUS) was used to measure perceived usability. It consists of 10 statements that assess users’ perceptions of system ease of use and overall usability [20,21]. Each statement is rated on a 5-point Likert scale, ranging from “strongly disagree” (1) to “strongly agree” (5), capturing both ease of use and learnability. SUS scores are calculated by first adjusting responses: for odd-numbered items, 1 is subtracted from the user’s rating, and for even-numbered items, the rating is subtracted from 5. The adjusted scores for each statement are summed, and the total is multiplied by 2.5 to convert the raw score to a range of 0 to 100. On the basis of empirical benchmarks reported by Bangor et al. [21], SUS scores above 68 are generally interpreted as above average, whereas scores around 80 or higher are commonly associated with excellent usability. These benchmarks provide a practical reference for interpreting system usability levels. The SUS has demonstrated strong psychometric properties across diverse systems and application domains, including high internal consistency and established construct validity. Prior validation studies have shown that SUS scores are robust and interpretable even in small-sample usability evaluations, making the instrument suitable for early-stage and pilot studies [20,21].

The User Experience Questionnaire (UEQ) evaluates multiple dimensions of perceived user experience, including *attractiveness, pragmatic quality*, and *hedonic quality* [22]. The UEQ consists of 26 items rated on a 7-point semantic differential scale ranging from −3 (most negative) to +3 (most positive), capturing users’ subjective impressions of different aspects of system interaction.

- Attractiveness: reflects the overall appeal of the system and represents users’ general impression.
- Pragmatic quality: captures perceived task-oriented aspects of the system use, focusing on how well users feel the system supports task accomplishment through three subdimensions: (1) perspicuity: ease of understanding and familiarization, (2) efficiency: perceived smoothness and effort associated with task execution, and (3) dependability: user’s perceived sense of control and predictability during interaction.
- Hedonic quality: captures the emotional and experiential aspects of interaction, covering (1) stimulation: how engaging and motivating the system feels; and (2) novelty: perceived originality and creativity of the system.

UEQ scale values above 0.8 are commonly interpreted as indicating a positive experience, whereas higher values may be classified as above average or excellent when compared against UEQ benchmark distributions, depending on the specific scale [23,24]. By distinguishing between pragmatic and hedonic qualities, the UEQ provides insight into both task-oriented interaction perceptions and experiential aspects of system use. The distinction is particularly relevant for AR systems, where perceived interaction support and user engagement jointly shape overall user experience. Validation studies of the UEQ have demonstrated acceptable to good internal consistency across its subscales and established construct validity for distinguishing between pragmatic and hedonic dimensions of user experience across a wide range of interactive systems [22-24].

The Technology Acceptance Model (TAM) assesses user acceptance of new technologies based on the relationship between two main dimensions: (1) perceived usefulness (PU), which measures the extent to which users believe that using a given technology enhances their job performance; and (2) perceived ease of use (PEU), which evaluates the extent to which users believe that using a technology will result in less effort to perform their tasks, focusing on its intuitiveness and the learning curve involved [25]. For this study, TAM was adapted to include 12 items across 2 primary dimensions, each rated on a 7-point Likert scale, ranging from “strongly disagree” (1) to “strongly agree” (7). Scores are averaged for each dimension. High scores across both dimensions suggest that users view the system as both beneficial and user-friendly—key factors for ensuring sustained use [26]. The PU and PEU constructs within TAM have demonstrated strong reliability and predictive validity for technology adoption and use intention across numerous information systems and health care technology studies, supporting their use in evaluating acceptance of emerging technologies, such as AR [25,26].

### Statistical Analysis

In this observational pilot study, there were no missing data for survey responses, and all analyses were descriptive in nature and aimed at characterizing perceived usability, user experience, and technology acceptance of the AR system across clinical roles. For each outcome measure, summary statistics were computed separately for the team leader and medication nurse roles. For the SUS, UEQ, and TAM measures, central tendency and variability were summarized using means and SDs. SEs and 95% CIs for the mean were calculated to indicate the precision of the estimates. Where appropriate, medians and IQRs were visualized using box plots to illustrate score distributions.

Given the small sample size and the exploratory nature of this pilot evaluation, no formal hypothesis testing or inferential comparisons between roles were performed. Instead, overlapping CIs were used to support cautious interpretation of observed differences, consistent with recommendations for early-stage usability and feasibility studies.

## Results

### Participant Demographics

A total of 10 health care professionals participated in the study, comprising 5 (50%) pediatric emergency physicians (team leaders) and 5 (50%) emergency nurses (medication nurses). Participants varied in age and clinical experience, with medication nurses generally reporting longer durations of practice and greater exposure to CA events. Most participants had limited prior experience with AR technologies, particularly in professional clinical contexts. Table 2 provides an overview of the participants’ demographic characteristics.

**Table 2.** Participant demographics.

	Team leader (n=5)	Medication nurse (n=5)
Gender, n (%)		
Male	3 (60)	1 (20)
Female	2 (40)	4 (80)
Age (y), median (IQR)	41 (40‐42)	45 (42‐46)
Duration in practice (y), median (IQR)	12 (11‐13)	20.5 (17‐24.25)
How many times have you had to care for a child in cardiac arrest during a *real, live event* in the past 2 y?, median (IQR)	1 (1‐4)	2 (2‐3)
How many times have you had to care for a child in cardiac arrest during a *simulated event* in the past 2 y?, median (IQR)	4 (2‐4)	5 (4‐6)
Have you ever used any type of augmented reality device for *professional use*?	1 participant with prior experience (>10 times)	No prior experience
Have you ever used any type of augmented reality device for *recreational use* (e.g., gaming)?	3 participants (>10 times)	1 participant (1‐4 times)

### Perceived Usability

**Figure 4.**
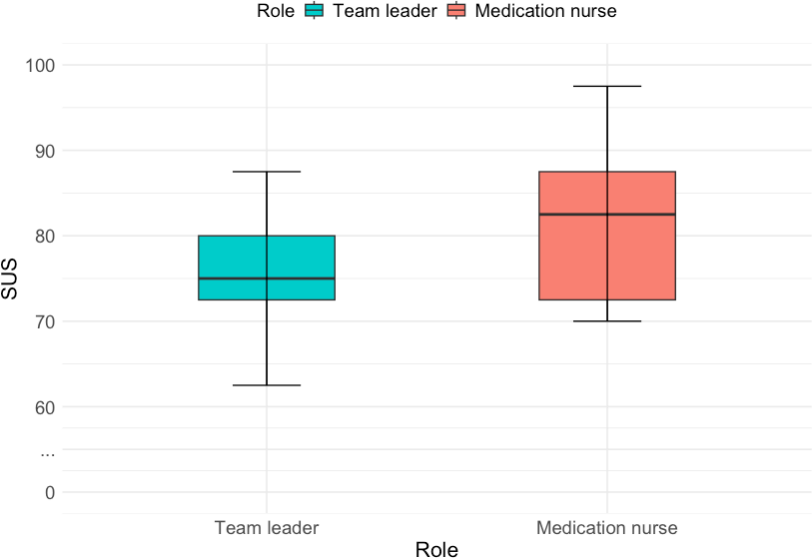
Box plot displaying System Usability Scale (SUS) scores for the team leader and medication nurse roles using the augmented reality support system. Higher median SUS scores for medication nurses indicate greater ease of interaction and workflow support, reflecting an “excellent” grade compared to the “good” usability rating for the team leader role.

The AR system demonstrated favorable perceived usability for both roles (Figure 4); however, the precision of these estimates varied across roles. The SUS revealed that the team leader role scored a mean of 75.50 (SD 9.25, SE 4.14, 95% CI 64.00-87.00), corresponding to a “B” grade (74.10‐77.10) on the SUS grading scale, categorized as “good” (Multimedia Appendix 2). The score generally suggests that team leaders perceived the system as usable and user-friendly; however, the relatively wide CI reflects uncertainty associated with the small sample size and indicates that this estimate should be interpreted cautiously.

The medication nurse role achieved a higher mean score of 82.00 (SD 11.20, SE 5.02, 95% CI 68.00-96.00), corresponding to an “A” grade (80.80‐84.00), which falls within the “excellent” usability range (Multimedia Appendix 2). Although the point estimate suggests a stronger perceived usability for medication nurses, the overlapping CIs between roles indicate that differences should not be interpreted as definitive in this pilot study. Overall, both roles reported favorable usability perceptions, with variability reflecting limited precision.

### Perceived User Experience

#### High-Level Results: Attractiveness, Pragmatic Quality, and Hedonic Quality

**Figure 5.**
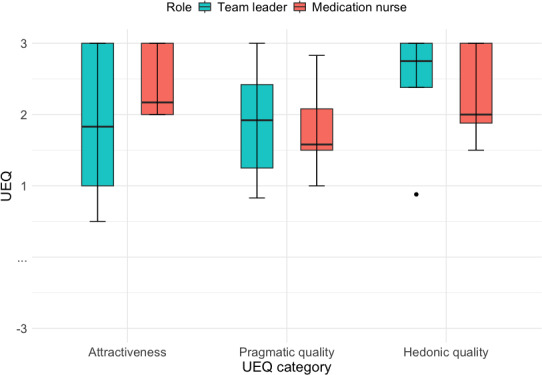
Comparison of User Experience Questionnaire (UEQ) scores—attractiveness, pragmatic quality, and hedonic quality—across roles. Both groups rated the system well above benchmark levels.

Perceived user experience was assessed using the UEQ, capturing participants’ subjective evaluations across attractiveness, pragmatic quality, and hedonic quality. The following results summarize mean scores and associated uncertainty for each dimension by clinical role (Figure 5).

- Attractiveness: The team leader role scored a mean of 1.87 (SD 1.14, SE 0.51, 95% CI 0.45-3.28), whereas the medication nurse role scored higher, with a mean of 2.43 (SD 0.52, SE 0.23, 95% CI 1.79-3.08). Both scores surpass the above-average benchmark, indicating a favorable overall impression of the system’s appeal, but the wider CI for team leaders indicates greater variability in perceived appeal (Multimedia Appendix 3).
- Pragmatic quality: Pragmatic quality scores were similarly positive across roles, with team leaders reporting a mean score of 1.88 (SD 0.87, SE 0.39, 95% CI 0.80-2.97) and medication nurses reporting a mean score of 1.80 (SD 0.69, SE 0.31, 95% CI 0.94-2.66). The overlapping CIs suggest comparable perceived task support.
- Hedonic quality: The team leader role scored a mean of 2.40 (SD 0.89, SE 0.40, 95% CI 1.30-3.50), whereas the medication nurse role scored a mean of 2.28 (SD 0.69, SE 0.31, 95% CI 1.42-3.13). These high scores highlight that users perceived the system as engaging and stimulating, contributing to a positive user experience, but the width of the CIs underscores the preliminary nature of these findings.

#### Pragmatic Quality Subdimensions

The analysis of pragmatic quality subdimensions (Figure S6 in Multimedia Appendix 1) revealed similar patterns across roles.

- Perspicuity: Both the team leader and medication nurse roles reported a mean score of 1.80. The team leader’s result (SD 1.14, SE 0.51, 95% CI 0.39-3.21) and the medication nurse’s results (SD 0.76, SE 0.34, 95% CI 0.86-2.74) indicated greater variability in perceived ease of learning among team leaders.
- Efficiency: For task completion speed and support, both roles achieved high mean scores of 2.15. The team leader’s score (SD 0.68, SE 0.30, 95% CI 1.31-2.99) and the medication nurse’s score (SD 0.38, SE 0.17, 95% CI 1.68-2.62) suggest perceived efficiency benefits, although precision remains limited.
- Dependability: The team leader role achieved a mean score of 1.70 (SD 1.02, SE 0.46, 95% CI 0.43-2.97), whereas the medication nurse role scored slightly lower at 1.45 (SD 1.30, SE 0.58, 95% CI −0.17 to 3.07). These scores indicate that users felt a good level of control (predictable), but the CI spanning zero indicates uncertainty regarding perceived control, highlighting this dimension as an area requiring further investigation.

#### Hedonic Quality Subdimensions

The analysis of hedonic quality subdimensions (Figure S7 in Multimedia Appendix 1) focused on stimulation and novelty, capturing the emotional and experiential aspects of user interaction with the AR system.

- Stimulation: The team leader role achieved a mean score of 2.25 (SD 0.94, SE 0.42, 95% CI 1.09-3.41), whereas the medication nurse role scored similarly at 2.20 (SD 0.84, SE 0.37, 95% CI 1.16-3.24). These scores suggest that the system is engaging and helps sustain users’ interest, motivating them throughout its use, but overlapping CIs and moderate width reflect limited precision in this pilot evaluation.
- Novelty: This subdimension assesses the system’s originality and innovative aspects. The team leader role scored a mean of 2.55 (SD 0.87, SE 0.39, 95% CI 1.47-3.63), whereas the medication nurse role scored a mean of 2.35 (SD 0.86, SE 0.38, 95% CI 1.28-3.42). These results indicate that users perceived the AR system as innovative, contributing to a unique and satisfying experience, but overlapping CIs again reflect limited precision.

### Perceived Technology Acceptance

**Figure 6.**
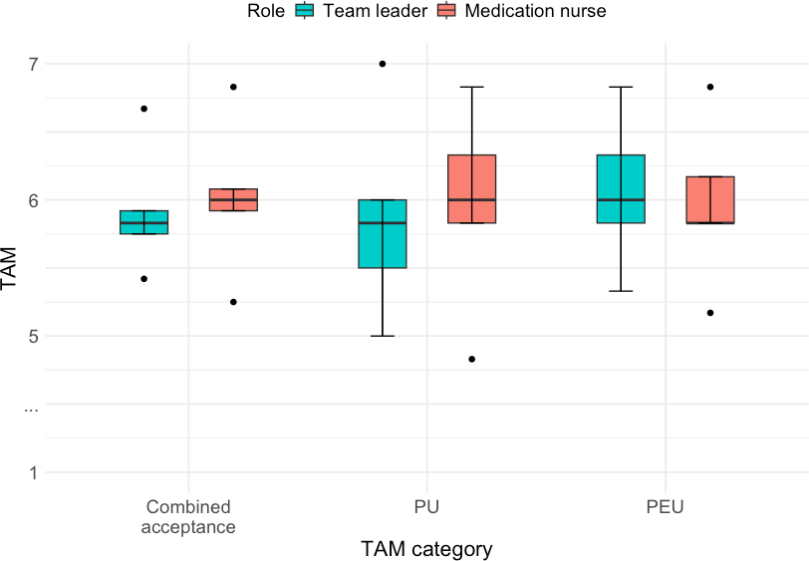
Technology acceptance model (TAM) results showing high perceived usefulness (PU) and perceived ease of use (PEU) for both roles, suggesting strong intention to adopt the augmented reality system in clinical training or practice.

The TAM scores were evaluated across PU and PEU. Combined scores were also calculated to provide an overall measure of acceptance for each role (Figure 6).

- Combined acceptance: The team leader role scored a mean of 5.92 (SD 0.46, SE 0.21, 95% CI 5.35-6.49), whereas the medication nurse role achieved a slightly higher score of 6.02 (SD 0.56, SE 0.25, 95% CI 5.32-6.71). The overlapping CIs suggest broadly comparable acceptance levels.
- PU: The team leader role achieved a mean score of 5.87 (SD 0.74, SE 0.33, 95% CI 4.95-6.78), whereas the medication nurse role scored slightly higher at 5.96 (SD 0.74, SE 0.33, 95% CI 5.05-6.88). While this indicates strong acceptance, CI width reflects uncertainty inherent to the small sample size.
- PEU: The team leader role scored a mean of 6.06 (SD 0.56, SE 0.25, 95% CI 5.37-6.76), whereas the medication nurse role scored similarly at 5.97 (SD 0.60, SE 0.27, 95% CI 5.22-6.72). These point estimates indicate strong acceptance, but CI width again reflects uncertainty.

## Discussion

### Summary of Main Findings

This study examined the feasibility and perceived usability, user experience, and acceptance of a role-specific AR decision support system designed for resuscitation team leaders and medication nurses. Consistent with the study objectives, clinicians generally perceived the system as usable, intuitive, and acceptable within a high-fidelity simulation context. Perceptions varied by role, reflecting differences in information needs, visual attention demands, and task responsibilities during CA management. These findings suggest that role-tailored AR interfaces are a potential tool for supporting cognitive work in resuscitation settings [15,27], while also underscoring that the present system represents an early-stage, proof-of-concept interface evaluated primarily through subjective measures.

### Interpretation of Findings and Relation to Prior Work

Across instruments assessing perceived usability, user experience, and technology acceptance, participants reported favorable impressions of the AR system. These results indicate that clinicians were able to understand and interact with the interface with minimal difficulty and perceived the system as appropriate for use in a simulated resuscitation workflow. Differences in perceived usability and acceptance between team leaders and medication nurses likely reflect role-specific cognitive and visual demands, as team roles in dynamic, safety-critical environments impose distinct situation awareness requirements and attentional burdens depending on task responsibilities and information density [28]. In particular, the team leader interface presented a higher density of information intended to support situational awareness and decision coordination, which may have contributed to comparatively lower—but still positive—perceptions of ease of use.

Participants’ responses suggest that the interface aligned with expectations for workflow support in emergent care contexts, where information must be rapidly accessible and interpretable at a glance. These findings are consistent with prior AR and mixed-reality research in clinical and safety-critical domains, which has shown that spatially anchored, role-relevant visual cues can be perceived as supportive when they reduce the need for external references and centralize task-critical information [29,30]. Importantly, these findings reflect perceived support rather than measured improvements in performance, workload, or coordination.

Several participants noted during postsimulation debrief discussions that the AR displays helped them maintain focus on the resuscitation process and reduced reliance on external reference materials. These observations represent subjective reflections elicited during informal debriefing rather than systematically collected performance data and should therefore be interpreted as experiential insights rather than evidence of objective benefit.

### Ease of Use, Learnability, and PU

High PEU and learnability indicate that clinicians felt they could quickly become comfortable with the interface, an important consideration for emergency contexts where training time is limited [31]. The visual organization of information, use of glanceable timers, and limited interaction complexity appeared to align with clinicians’ expectations for decision support during resuscitation [28,29].

Clinicians also viewed the system content as relevant and supportive of their respective roles, as reflected in ratings related to PU and pragmatic quality. These perceptions are consistent with the underlying design rationale of emphasizing medication-specific information for nurses and algorithmic pathway cues for team leaders. Although prior research suggests that highly usable systems can reduce cognitive load and support more fluid task execution [27], such perceptions should not be interpreted as evidence of improved task performance, guideline adherence, or efficiency. None of these outcomes were directly measured in the current study, and future evaluations must incorporate objective task-level metrics to determine whether perceived utility translates into measurable clinical benefits.

### Novelty, Engagement, and Hedonic Experience

Participants rated the AR system highly on hedonic quality dimensions—novelty and stimulation—indicating that the interface was perceived as original, engaging, and distinct from existing tools. These responses reflect perceived innovativeness and experiential engagement rather than satisfaction or effectiveness. Such hedonic responses are encouraging for simulation-based training contexts, where engagement can influence motivation and willingness to adopt new tools [23]. Especially in AR, prior research demonstrated that spatially registered visual cues can increase engagement and perceived control [15,18,19].

At the same time, novelty effects are well documented in evaluations of emerging technologies, particularly during short-term exposure. Perceptions of engagement and stimulation may change with repeated use or prolonged deployment, emphasizing the need for longitudinal studies to assess sustained acceptance and experiential quality over time.

### Role-Specific AR Design Implications

A central contribution of this study is the identification of actionable design principles for AR support during CA resuscitation. The iterative prototyping process revealed that AR interfaces should prioritize role-relevant information to minimize unnecessary visual load, use dominant and easily glanceable timers for actionable intervals such as CPR cycles and epinephrine dosing, maintain algorithmic transparency to allow clinicians to view the full pulseless arrest algorithm, and organize spatial layouts clearly by separating medication instructions, procedural steps, and timing cues. These principles provide practical guidance for developers of future AR support tools. While these design choices were intended to support coordination, anticipation, and situational awareness, their operational impact on team performance and guideline adherence remains to be empirically evaluated in future studies. These design considerations align with prior work on situation awareness, cognitive aids, and role-specific information presentation in safety-critical and resuscitation contexts [28-30].

### Real-World Implementation Considerations

Although the system achieved promising perception-based results in a controlled simulation environment, translating AR decision support into real clinical workflows presents substantial challenges. Cost, hardware maintenance, device sterilization, and user training remain key considerations for AR deployment in clinical settings [13,30]. Furthermore, seamless interoperability with existing electronic health record systems, secure handling of patient data, and efficient user training are essential for successful integration. Although none of our participants reported discomfort related to the headset bulkiness or fatigue, future iterations should explore lightweight, cost-effective head-mounted devices and web-based synchronization frameworks that ensure data security and workflow continuity. Addressing these implementation barriers will be critical to realizing the clinical impact of AR-based decision support systems. Given that the current evaluation involved standardized scenarios, conclusions about clinical applicability should be viewed as preliminary.

### Limitations and Future Work

While the AR system demonstrated high usability, user experience, and technology acceptance, several limitations should be acknowledged. The most notable limitation is the small sample size (n=10), which restricts statistical generalizability and inferential power. Participants had prior exposure to an early prototype, which may introduce some bias in perceived usability and novelty but also provide more implementation-focused feedback due to their familiarity with the system. Future studies will distinguish between first-time and repeat users to maintain objectivity.

This study was designed primarily to assess initial technical and interaction viability and user experience rather than to test hypotheses or perform comparative statistics. Accordingly, future formal evaluations with larger and more diverse participant samples are planned to validate reproducibility and strengthen external validity. The current evaluation also relied primarily on subjective self-report measures. Incorporating objective performance metrics—such as time to defibrillation, time to epinephrine administration, adherence to CPR cycles, and error frequency—will be crucial in future work. These indicators, combined with physiological or behavioral measures (e.g., eye-tracking, gaze-based workload assessment, or speech-based coordination analysis), can provide richer evidence for the system’s real-world effectiveness in improving team performance and reducing cognitive load. Additionally, the study’s simulated pediatric CA scenario, while useful for evaluation, may not capture the full range of real-world situations that resuscitation teams might encounter. Expanding the system’s evaluation to include a broader range of scenarios could improve its generalizability across diverse clinical environments.

To address these limitations, future research will involve testing the AR system in various CA simulation scenarios to assess its adaptability and reliability before clinical implementation. No major hardware stability issues were observed during testing, and participants, including those wearing corrective glasses, were able to use the device comfortably. Nonetheless, extended use may cause mild visual fatigue or vertigo in a small subset of users, as reported in prior AR literature [27], which warrants monitoring during longer clinical sessions. Plans include conducting an international multisite study with a larger, more diverse participant pool to gain broader insights. This study will also involve incorporating the AR tool into an expanded CPR support system, including additional tools such as a widescreen display for team information visualization, a tablet-based progress monitoring tool providing real-time clinical data, and advanced control interfaces. To gain deeper insights into user performance and behavior, follow-up studies will incorporate objective performance metrics, such as task completion time, gaze tracking, and speech analysis. These metrics will be instrumental in evaluating the system’s effectiveness in real-world, high-stakes environments, with the ultimate goal of refining and enhancing its role-specific support functionalities for future clinical use.

### Conclusions

This study demonstrates the feasibility and favorable perceived usability, user experience, and acceptance of a role-specific AR decision support system designed for pediatric resuscitation team leaders and medication nurses. Clinicians perceived the system as intuitive, clear, and appropriately tailored to their roles, supporting its potential use in simulation-based training and early-stage clinical exploration. Importantly, the present findings are limited to perception-based outcomes and do not provide evidence of improved performance, workload reduction, or guideline adherence. Rather, this work establishes a foundation for future evaluations that integrate objective measures and assess real-world impact. More broadly, the study illustrates how role-specific AR interfaces can be systematically designed and formatively evaluated as cognitive aids in high-stakes, team-based health care settings.

The innovation of this work lies in its explicit focus on role-specific, in-view AR decision support, which differs from prior studies that primarily evaluated role-agnostic cognitive aids delivered via tablets, posters, or nonadaptive AR displays. By empirically examining clinicians’ perceptions across distinct team roles, the study contributes early evidence and practical design guidance for developing role-aware AR interfaces aligned with differing cognitive demands and workflows. In real-world contexts, such role-tailored AR systems may inform the design of next-generation simulation training tools and guide the integration of wearable decision support into clinical resuscitation environments, contingent on future validation using objective performance metrics.

## Supplementary material

10.2196/72013 Multimedia Appendix 1 Supplementary figures illustrating augmented reality (AR) interface design prototypes, system architecture and control interfaces, and usability evaluation results for the role-specific AR decision support system.

10.2196/72013 Multimedia Appendix 2 Grading scale for System Usability Scale scores with corresponding percentile ranges, usability adjectives, and acceptability levels [32].

10.2196/72013 Multimedia Appendix 3 Interpretation criteria for User Experience Questionnaire scores across different scales [33].

## Abbreviations

ACH: Alberta Children’s Hospital
AR: augmented reality
CA: cardiac arrest
CPR: cardiopulmonary resuscitation
PEA: pulseless electrical activity
PEU: perceived ease of use
PU: perceived usefulness
SUS: System Usability Scale
TAM: technology acceptance model
UEQ: User Experience Questionnaire
UI: user interface
