# Supplementary material for: Perceived Usability, User Experience, and Technology Acceptance of Role-Specific Augmented Reality Decision Support Tools for Cardiac Arrest Resuscitation: Prospective Observational Pilot Study
Source: JMIR XR Spat Comput. 2026 Apr 7;3:e72013. doi: 10.2196/72013 (PMC13202500; doi:10.2196/72013)
Supplement: Multimedia Appendix 1 [file xr-v3-e72013-s001.docx]

## Multimedia Appendix 1. Additional Figures


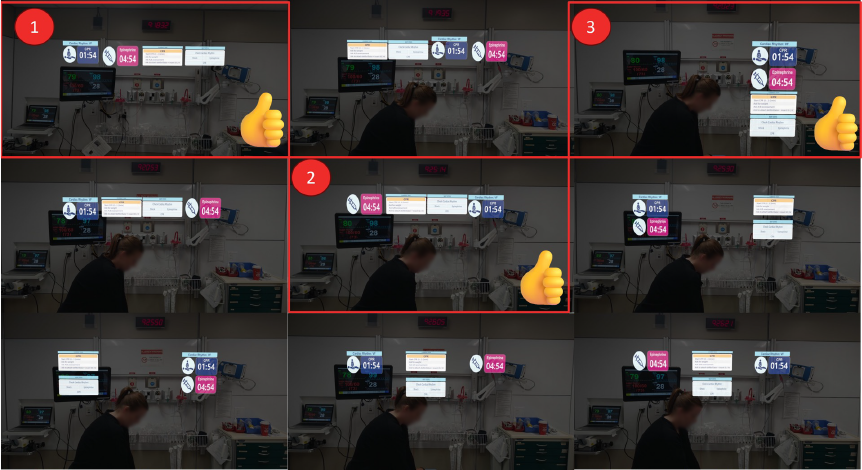


Figure S1. Static layout prototypes for team leaders. Three layout options (1–3) with thumbs-up icons indicate the designs selected for further development.


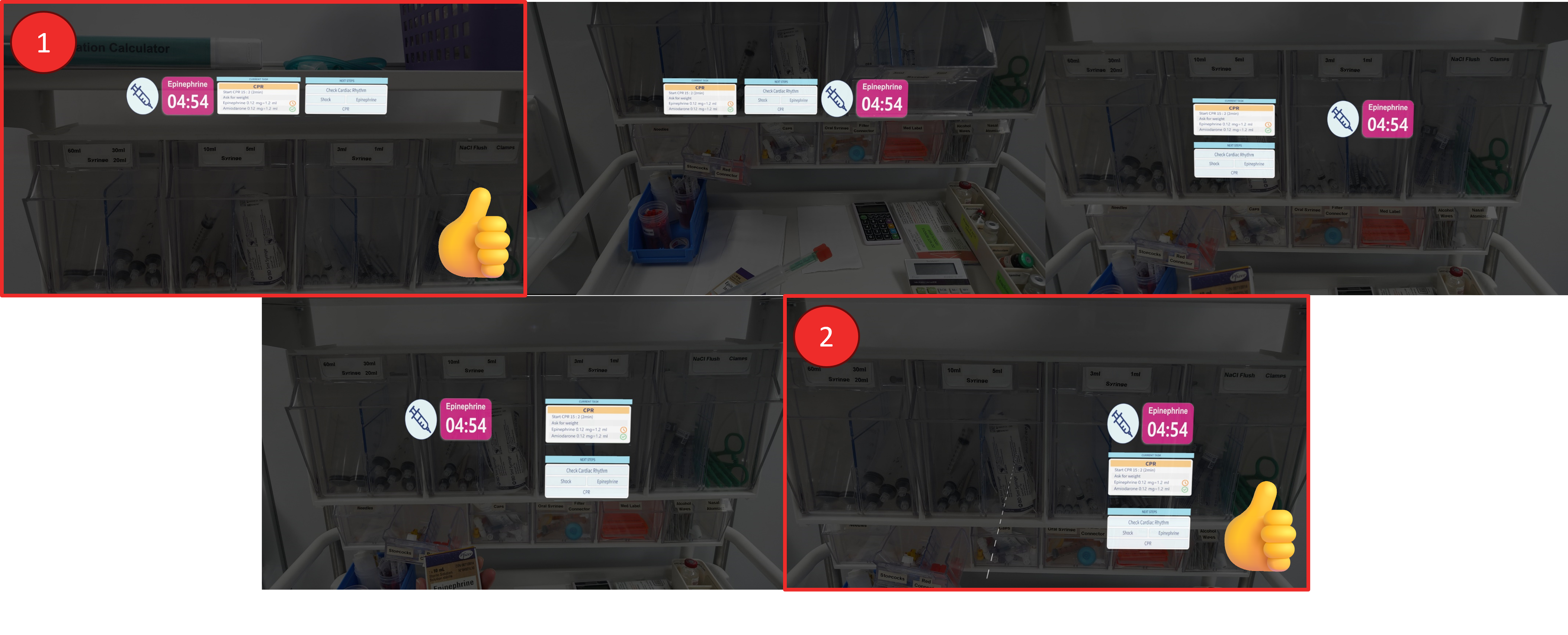


Figure S2. Static layout prototypes for medication nurses. Two layout options (1–2) with thumbs-up icons indicate the designs selected for further development.


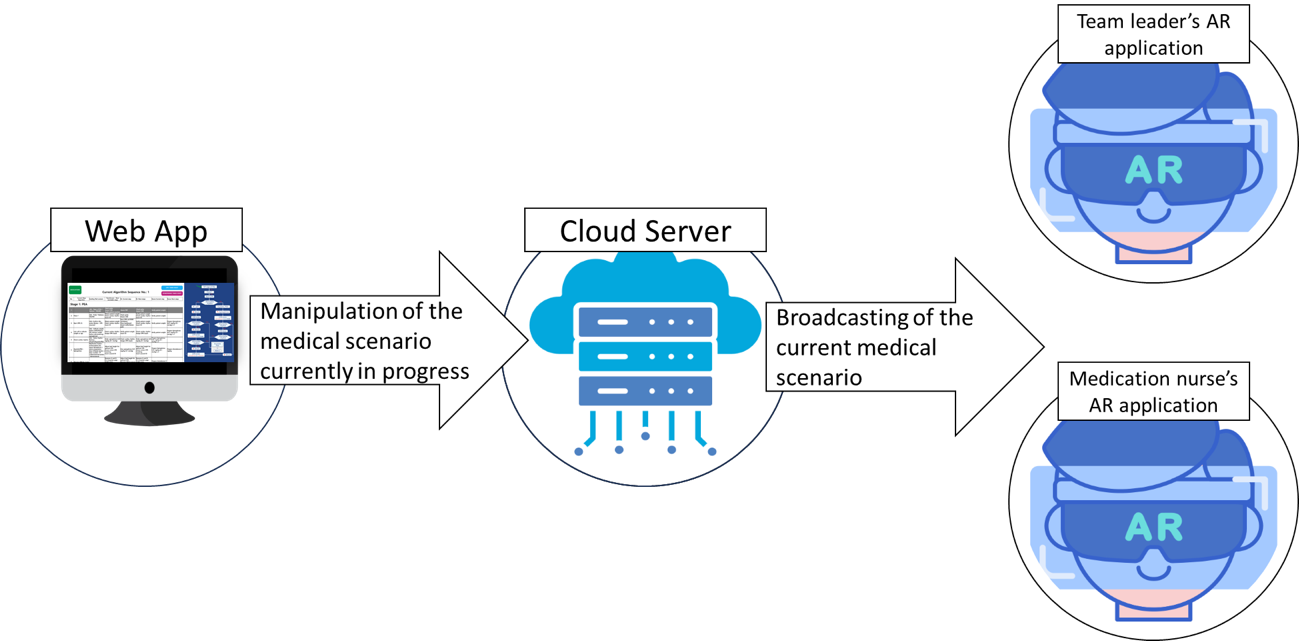


Figure S3. Overview of the AR system architecture illustrating the web-based control system, cloud server, and role-specific AR interfaces for the team leader and medication nurse.


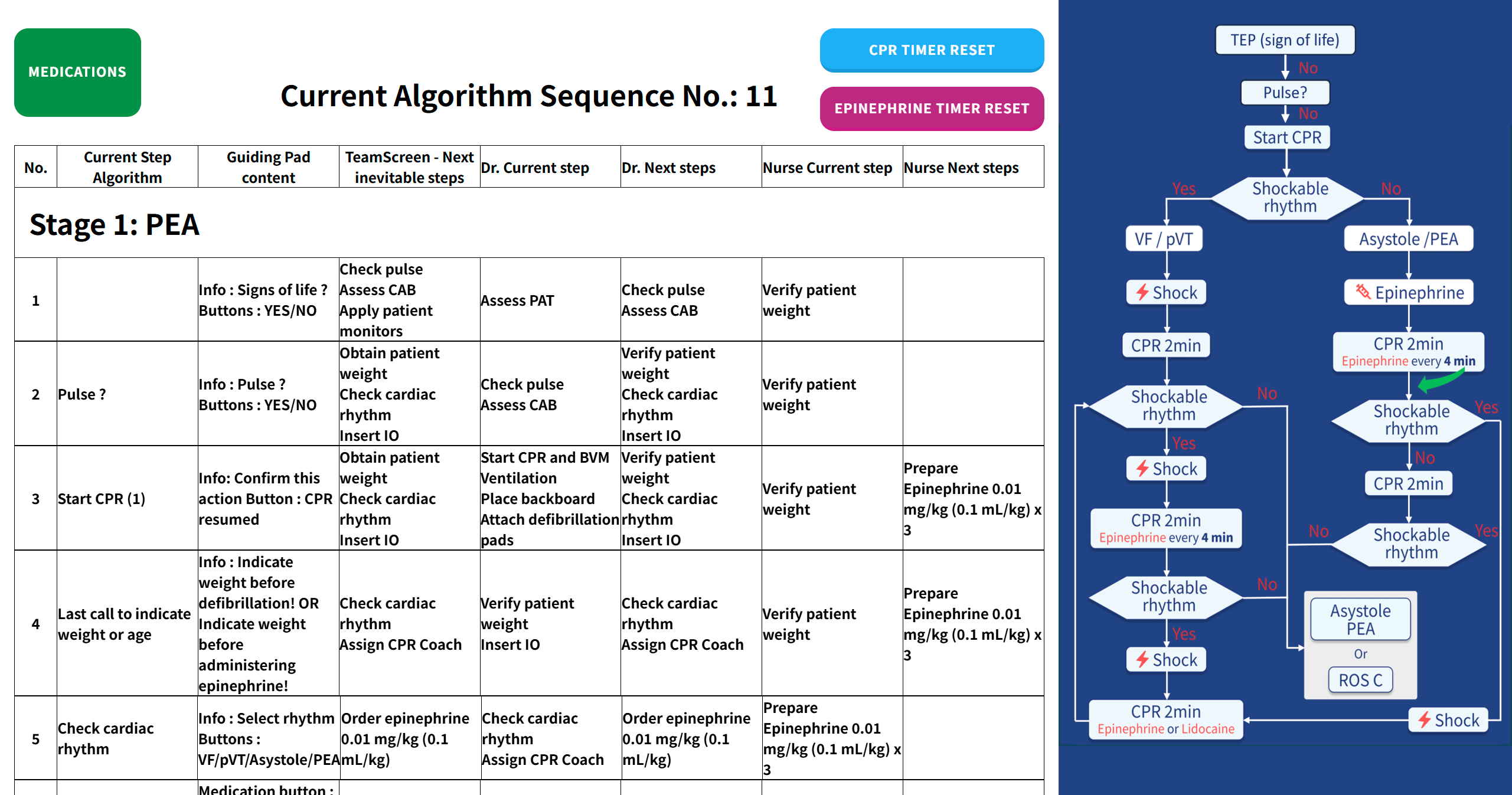


Figure S4. Web-based control application used by the experimenter to trigger stage transitions, medication alerts, and timer synchronization.


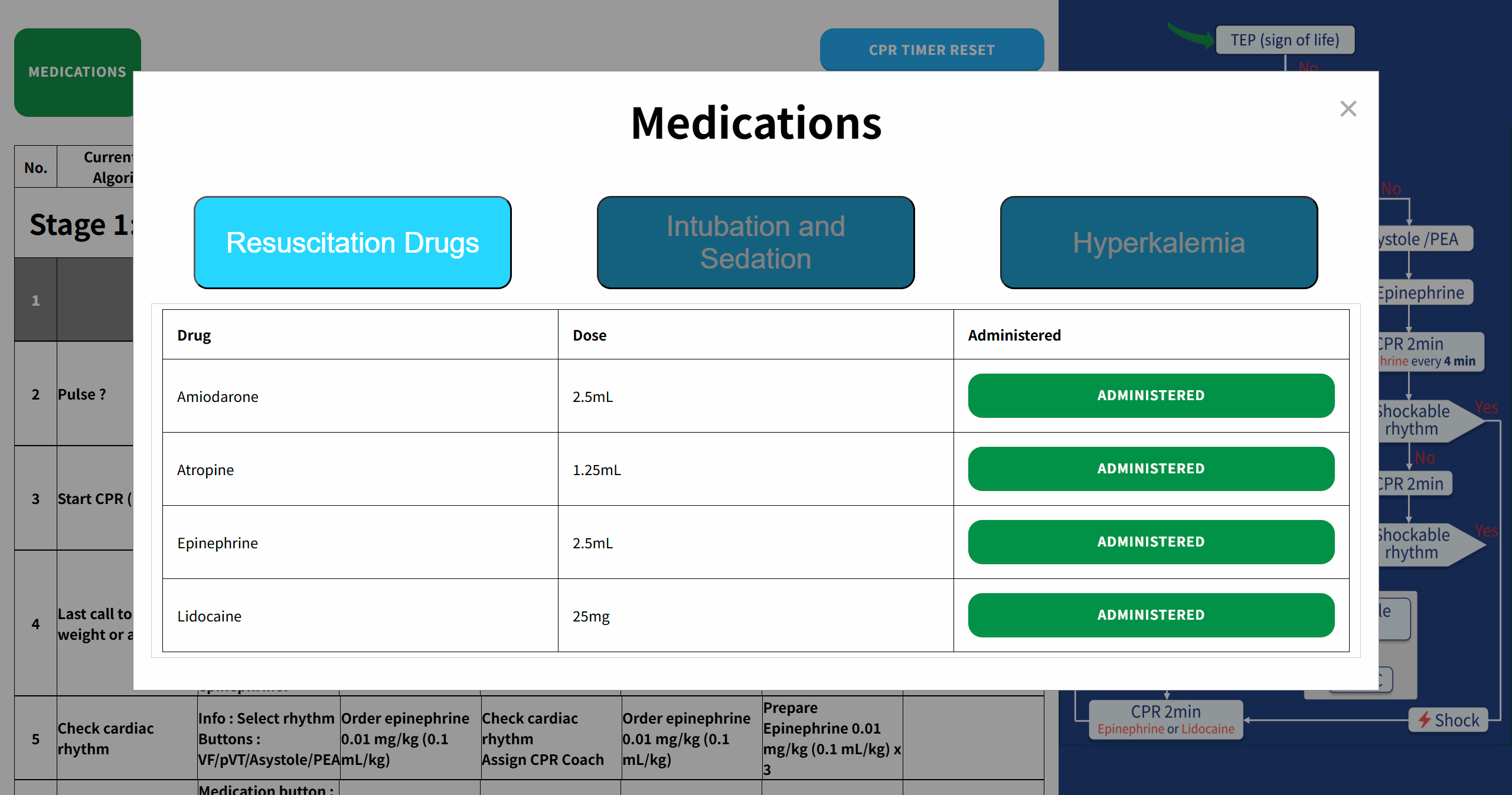


Figure S5. Medication control panel in the web application. Pressing “ADMINISTERED” synchronizes status with both AR interfaces.


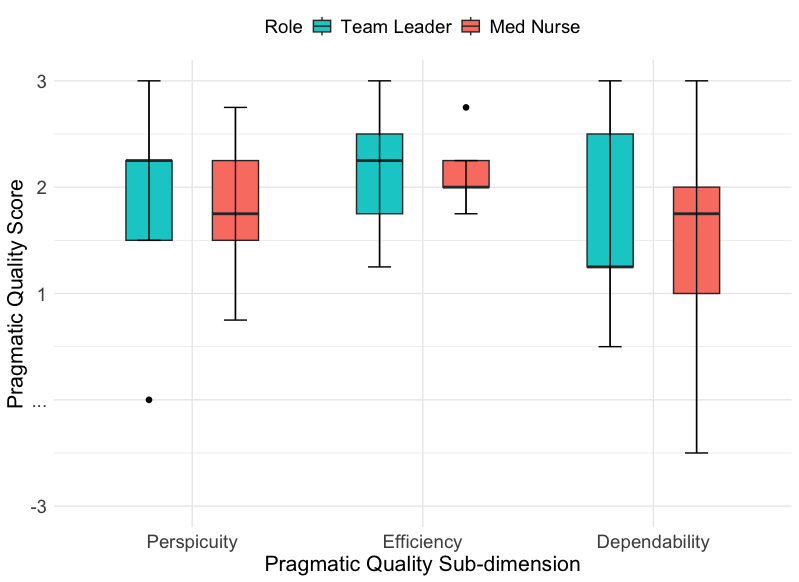


Figure S6. Box plot displaying pragmatic quality sub-dimensions (perspicuity, efficiency, dependability) for team leader and medication nurse roles, reflecting usability-focused aspects of the AR system.


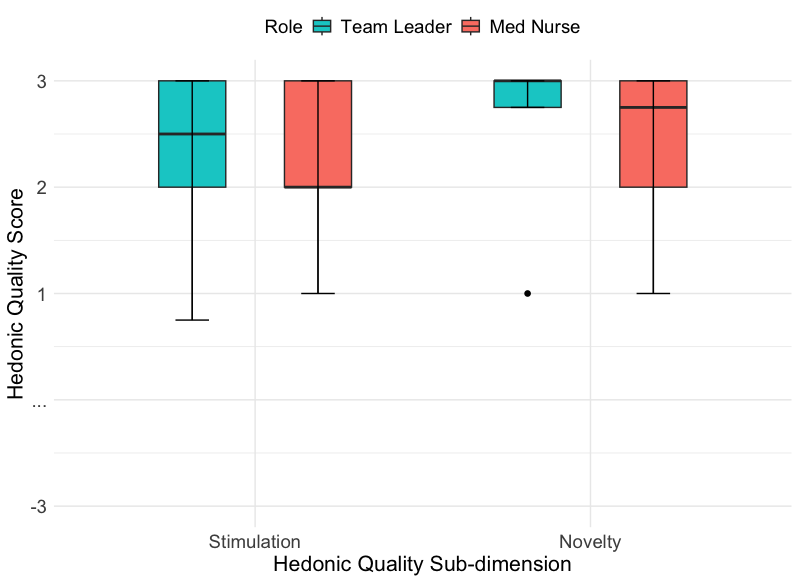


Figure S7. Box plot displaying hedonic quality sub-dimensions (stimulation and novelty) for team leader and medication nurse roles, representing the emotional and experiential user engagement with the AR system.
